# A case of lymphangioleiomyomatosis associated with endometrial cancer and severe systemic lupus erythematosus

**DOI:** 10.1186/s12885-016-2413-z

**Published:** 2016-07-04

**Authors:** Kensuke Suzuki, Kazunori Nagasaka, Katsutoshi Oda, Hiroyuki Abe, Daichi Maeda, Yoko Matsumoto, Takahide Arimoto, Kei Kawana, Masashi Fukayama, Yutaka Osuga, Tomoyuki Fujii

**Affiliations:** Department of Obstetrics and Gynecology, Faculty of Medicine, The University of Tokyo, Tokyo, Japan; Department of Pathology, Faculty of Medicine, The University of Tokyo, Tokyo, Japan

## Abstract

**Background:**

Lymphangioleiomyomatosis (LAM) is a rare idiopathic disorder that occurs in women of childbearing age, and consists of a diffuse proliferation of abnormal smooth muscle cells along the thoracic and abdominal lymphogenous route.

**Case presentation:**

We experienced a case of a 47-yo woman with recent history of systemic lupus erythematosus (SLE) diagnosed with endometrial cancer, initially suspected to have metastasized to pelvic and para-aortic lymph nodes based on preoperative diagnostic imaging. Subsequent pathological diagnosis revealed stage IB endometrial cancer without evidence of lymph node involvement. Instead, enlarged pelvic and para-aortic lymph nodes were found to be due to extrapulmonary LAM, from a primary lesion found inside the uterine myometrium. SLE improved after surgery.

**Conclusion:**

This is the first reported case of comorbid endometrial cancer, SLE, and aggressive LAM metastasizing to regional lymph nodes, and strengthens the clinical evidence for a common role of mTOR pathway hyperactivity and estrogen responsiveness in the pathophysiology of metastasizing lesions of the genital tract.

## Background

Lymphangioleiomyomatosis (LAM), a rare disorder of unknown incidence and prevalence, is characterized by abnormal proliferation of smooth muscle cells in the family of perivascular epithelioid cells (PEC) expressing human melanin black (HMB)-45. Estimated to affect up to 34 % of women with tuberous sclerosis complex (TSC) [1], the lung is the most common site of involvement, though extra-pulmonary LAM has been reported in various other systems including gynecologic organs. Although the precise cause of LAM remains unclear, its etiology has recently been genetically linked to well-characterized autosomal mutations of variable penetrance in *TSC1* or *TSC2* genes, as well as estrogen (E2) responsiveness [2–4]. In the female genital tract, LAM primarily affects the uterus [5–11], often mimicking malignant diseases [12]. Despite several studies reporting the usefulness of imaging for detection of lymph node disease for suspected metastasis [13–15], imaging findings in sporadic LAM without accompanying TSC are difficult to interpret prior to systematic surgical staging, leading to incorrect diagnoses in the absence of surgical resections.

In this report, we describe the case of a woman with SLE, found to have uterine LAM with lymphadenopathy, incidentally found on lymphoadenectomy for treatment of advanced uterine endometrial cancer. To the best of our knowledge, this is the first report of LAM with SLE and endometrial cancer in a single patient. This case report strengthens the possibility that mTOR abnormalities may trigger a variety of autoimmune and developmental disorders in women.

## Case presentation

### Case report

A 47-year-old Japanese woman, gravida 2, para 0, was referred to our institution for abnormal uterine bleeding. She had an extensive past medical history which included SLE, anti-phospholipid antibody syndrome (APS), idiopathic thrombocytopenic purpura (ITP), as well as previous dissecting right vertebral artery aneurysm and left cerebellar infarction. Her body mass index (BMI) was 18.1. Workup revealed severe anemia requiring an 8-unit red cell transfusion, and subsequent to gynecological examination, endometrial sampling cytology with conventional biopsy revealed Grade 1 endometrioid adenocarcinoma; serum tumor markers were obtained for evaluation and anticipated ongoing management of endometrial cancer; CA-125, CA19-9, and CEA levels were 176 U/mL, 27U/mL, and 1.8 ng/mL, respectively. Given her intravascular hypercoagulability due to ITP, we administered continuous heparin along with methylprednisolone (mPSL) pulse therapy to bring her hematology profile within an acceptable range of tolerability for surgery. The patient’s laboratory results and serologies are summarized in Table 1. Her medications included prednisolone 20 mg twice a day, candesartan 8 mg daily, amlodipine 5 mg daily, rabeprazole sodium 3 g daily, and 600 μg subcutaneous teriparatide daily.

**Table 1:** Laboratory results and serologies at the first medical examination

WBC	3.3×10^3^/μL	PT %	90.0 %	RF	6 IU/mL
Hb	9.1 g/dL	PT-INR	1.05	CH50	33.7 U/mL
Plt	2.2×10^4^/μL	APTT	62.0 s	SS-A	240.0 U/mL
		FDP	7.0 μg/mL	C3	58 mg/dL
Alb	3.3 g/dL	D-dimer	4.3 μg/mL	C4	11 mg/dL
LDH	319U/L			DS-DNA	0.9 IU/mL
BUN	24.1 mg/dL	CEA	1.8 ng/mL	SS-DNA	2.2 U/mL
Cre	0.85 mg/dL	CA19-9	27 U/mL	Antinuclear Ab	+
Na	139 mEq/L	CA125	176 U/mL	Lupus AC	2.67
K	4.2 mEq/L	CA15-3	12 U/mL	Anti CL-IgG	79 U/mL
Cl	105 mEq/L	NSE	11.0 ng/mL		
AST	19 U/L	SLX	25.0 U/mL	ESR	48
ALT	23 U/L	SCC	2.1 ng/mL		
CRP	0.29 mg/dL	CA72-4	4.4 U/mL		

**Fig. 1:**
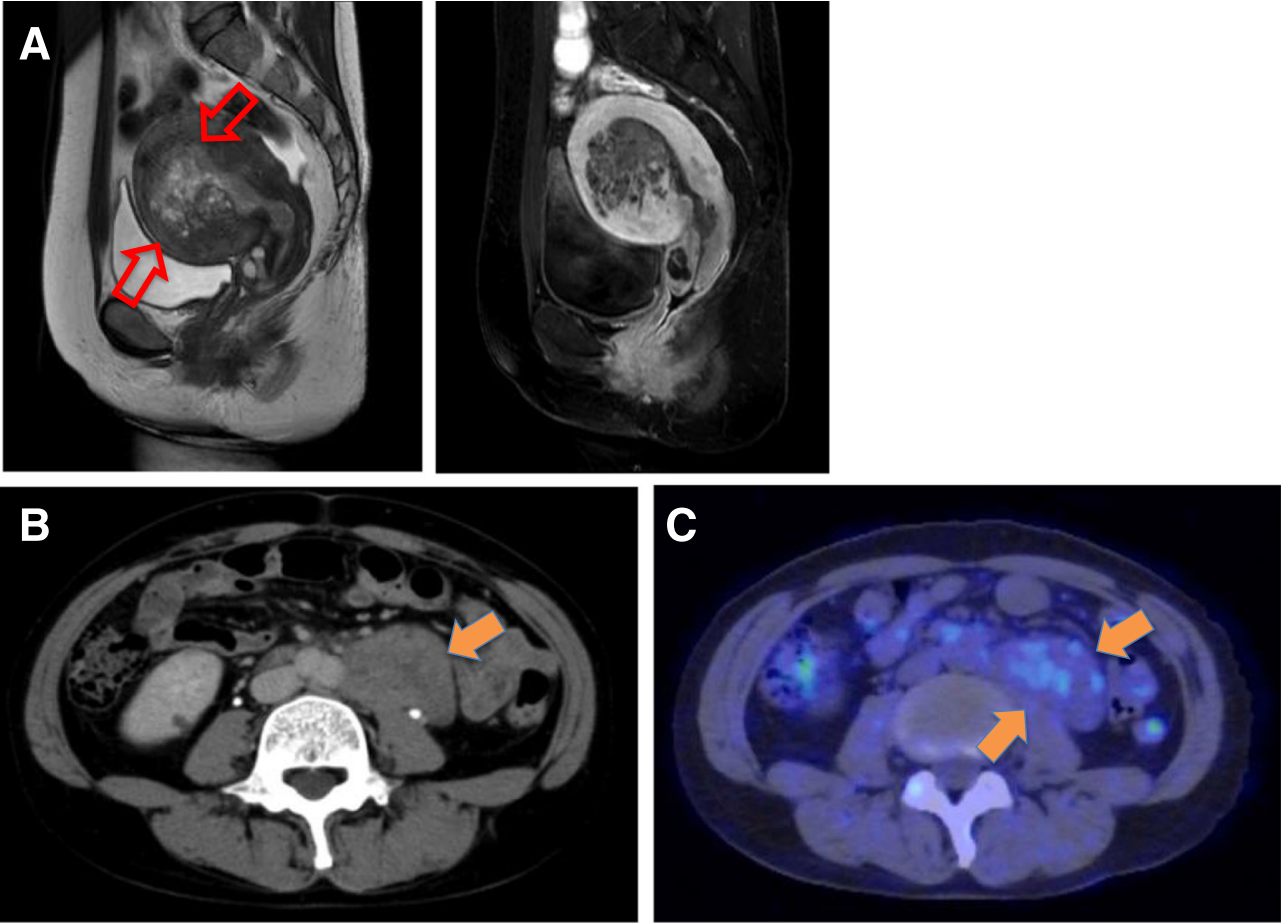
**a** Axial preoperative T2-weighted contrast magnetic resonance imaging (MRI) shows endometrium thickness in the uterine body. It has been diagnosed as Endometrial cancer of stage IB due to more than 50 % depth of myometrial invasion (*red arrowhead*). **b** The positron emission tomographic (PET) with CT scan. **c** shows pathologically elevated glucose metabolism in enlarged paraaortic lymph nodes (*orange arrowhead*)

Magnetic resonance imaging (MRI) studies revealed cancer invasion into the uterine myometrium (Fig. 1a). On computed tomography (CT) imaging, abnormal masses involving a large segment of multiple encapsulated lymphadenopathies and measuring up to 6 cm were present both in pelvic and para-aortic lymph nodes (Fig. 1b), strongly suggesting retroperitoneal metastases from endometrial cancer. However, positron emission tomography-computed tomography (PET-CT) imaging showed only slightly abnormal FDG uptake in the lymph nodes (Standardized uptake value (SUV)-Max = 2.1) (Fig. 1c) in comparison with high FDG uptake in the uterus (SUV-Max = 19.1). Eventually, total hysterectomy and bilateral salpingo-oophorectomy was performed for primary staging, avoiding lymph node dissection due to ongoing ITP-related thrombocytopenia.

**Fig. 2:**
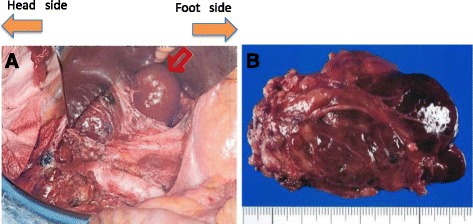
**a** Intraoperative photo showing the swollen paraaortic lymph nodes left along the aortic vessels (*red arrowhead*). **b** The excised maximum lymph node, approximately 7 cm in size, was solid and tender characteristic tumor

On the basis of preoperative findings, we initially diagnosed the patient as International Federation of Gynecology and Obstetrics (FIGO) stage IIIC2 uterine endometrial cancer, with 70 % myometrial invasion, lymphovascular invasion, and metastasis to the pelvic and para-aortic lymph nodes. The patient subsequently underwent adjuvant chemotherapy with paclitaxel (175 mg/m2) and carboplatin (area under the curve, 6). However, no change in size of lymphadenopathy was observed after 3 cycles of chemotherapy. As her overall condition improved, with platelets stabilizing at >80,000/μL, and the patient wishing to avoid radiation therapy due to the considerable complications, we then performed dissection of retroperitoneal (pelvic and para-aortic) lymph nodes. On gross exam, we found well-circumscribed lymph node masses growing along the lymph vessels (Fig. 2a), which were systematically dissected (Fig. 2b), and found to have no metastatic involvement or cured remnants of metastatic disease (0 out of 100 lymph nodes), although there was an option to perform intraoperative diagnosis using frozen sections if necessary. Final pathological diagnosis was consistent with LAM arising from the retroperitoneal lymph nodes.

**Fig. 3:**
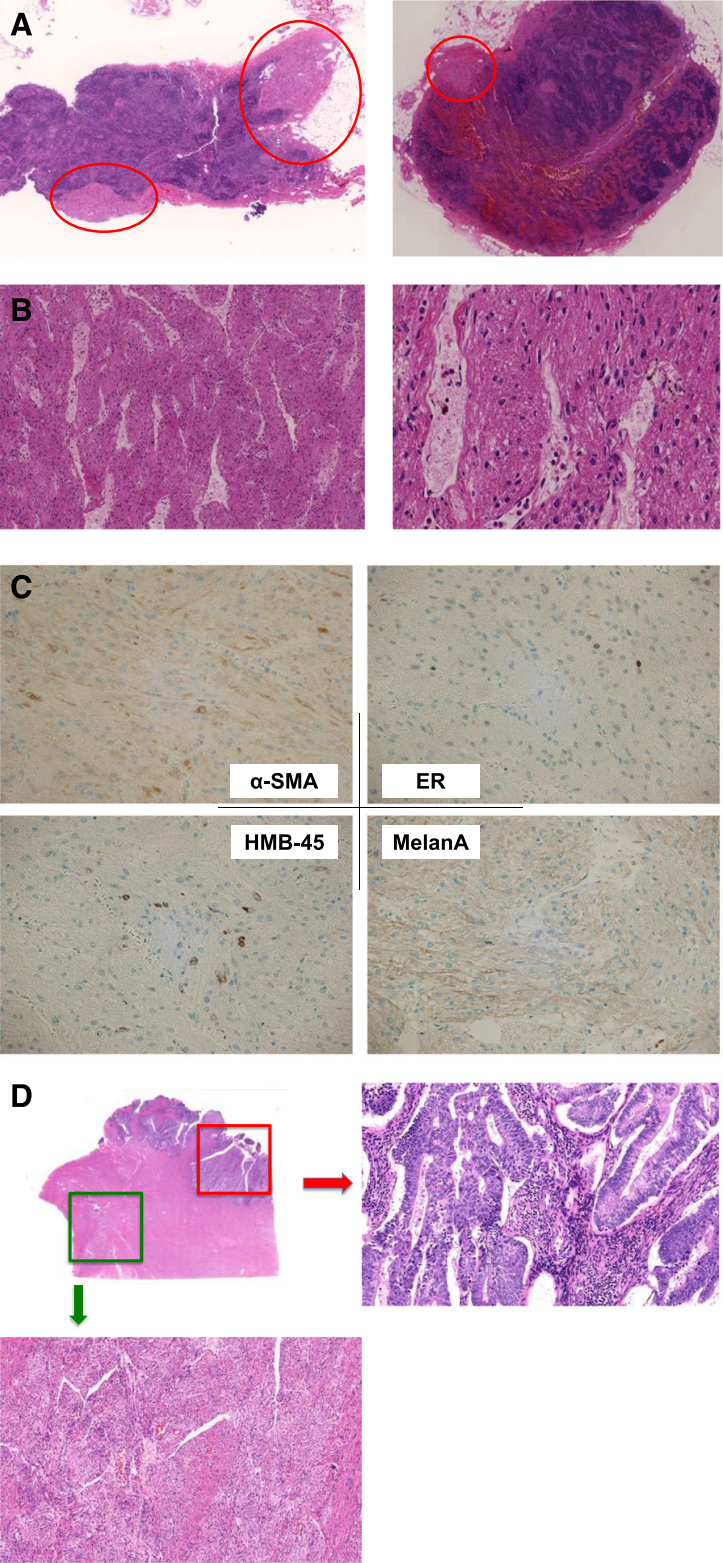
**a** The maximum size lymph node was dissected from the left lateral aortic lesion. Microscopically, masses was composed of neoplastic smooth muscle leiomyoma-like tumor cells with clear to eosinophillic cytoplasm arranged in alveolar pattern without necrosis. **b** Slit-like vascular channels lined by endothelial cells were seen in the lymph node. The cells with irregular shaped nucleus were arranged in nested pattern. **c** Lymph node tumors were focally positive for smooth muscle actin (SMA), caldesmon, Melan A, HMB-45, and ER, characteristics suggestive of LAM. Estrogen receptor (ER) was also focally positive. **d** Spindle cell proliferation suggestive of regional LAM tissue was seen in the re-examination of uterine tissue from the primary operation

Microscopically, masses was composed of neoplastic smooth muscle leiomyoma-like tumor cells with clear to eosinophillic cytoplasm arranged in alveolar pattern without necrosis (Fig. 3a), and slit-like vascular channels lined by endothelial cells (Fig. 3b). Lymph node tumors were focally positive for smooth muscle actin (SMA), caldesmon, Melan A, HMB-45, and estrogen receptor (ER), characteristics suggestive of LAM (Fig. 3c). Interestingly, re-examination of uterine tissue from the primary operation revealed that regional LAM tissue was co-localized with endometrial cancer with similarly arranged alveolar structure (Fig. 3d). Unlike normal myometrium smooth muscle cells, immunostaining of smooth muscle cells for HMB-45 and Melan A was positive in the LAM lesions. Moreover, we found that LAM cells had locally invaded into the lymphatic vessels of the myometrium. Taken together, our findings presented herein suggest that LAM initially occurred in uterine smooth muscle, subsequently invading the retroperitoneal lymph nodes by ascending via a lymphogenous route. The patient is currently asymptomatic after the final diagnoses of LAM and endometrial cancer (stage IB), with no evidence of recurrence or metastasis to date. No recurrent enlargement of lymph nodes or pulmonary LAM has been observed on repeat CT imaging. However, she is currently maintained on low dose warfarin therapy for SLE-related intravascular hypercoagulability. Long-term follow-up by both internal medicine and gynecological healthcare providers will continue to be important to correctly diagnose LAM and/or cancer-related diseases.

## Discussion

LAM is an idiopathic and intractable disease predominantly affecting females of childbearing age. Classified into pulmonary and extrapulmonary types, most patients are identified by onset of pulmonary complications, such as respiratory failure after pneumothorax [1]. Sporadic extrapulmonary LAM remains an unusual diagnosis in the clinical setting [5]. No previous publication has reported on its association with both SLE and endometrial cancer, and this unique case suggests a possible common etiology involving dysfunction of cell regulation functions of the mTOR pathway.

As noted above, the loss of function mutations identified in TSC1 and TSC2, have been broadly detected in pulmonary LAM cells, and likely explains the high correlation found between pulmonary LAM and TSC [16]. These TSC1/TSC2 loss of function mutations activate mammalian target of rapamycin (mTOR) protein kinases, which promote both cell proliferation and survival, have been implicated in various types of cancer [17]. Recently, the mTOR pathway has been found to play a crucial role in the development of endometrial cancer with high frequency of mutations in *PTEN* and/or *PIK3CA* [18]; knowledge about genetic alterations involved in this pathway offer potential treatment strategies. Furthermore, constitutive activation of the mTOR pathway has recently been reported in SLE [19]. Taken together, the four diseases (LAM, TSC, SLE, and endometrial cancer) appear to share a robust association with mTOR pathway activation. It is noteworthy that no particular risk factors for endometrial cancer were found in her personal or family history. Therefore, it is a reasonable assumption that our patient’s history of SLE may be causally related to both LAM and endometrial cancer. Though we have not yet performed genetic testing for germline mutations to reveal an association with mTOR pathway deregulation that may cause TSC, the presence of severe SLE, highly suggestive of constitutive hyperactivation of mTOR signaling, may lead to endometrial cancer as well as particularly aggressive LAM cells present in uterine myometrium and lymphogenously invading the retroperitoneal lymph nodes.

## Conclusion

Clinicians should also be aware of a previous report suggesting that extrapulmonary uterine LAM may precede both TSC and pulmonary LAM by several years [4, 6], a reasonable finding in the setting of ongoing and unremitting mTOR hyperactivity and estrogen responsiveness. As such, we anticipate that this patient will continue to be at risk for pulmonary LAM in the future. In Japan, there have been several reported cases of pulmonary LAM recurrence even after lung transplant. For treatment of LAM, gonadotropin releasing hormone agonists or progesterone therapy has been used for decades, though their effectiveness remains controversial and definitive treatment remains elusive. Recently, sirolimus, an mTOR inhibitor, has been investigated as a potential therapeutic agent [20]. As a previous study has described pulmonary LAM discovered a decade after initial diagnosis of a gynecologic lesion [5], novel therapies may play a role in both treatment as well as prevention. Based on currently available evidence, we feel that sirolimus on gynecologic field should be used with caution, and considered only after thorough assessment and in conjunction with scheduled imaging surveillance for recurrent disease.

As mentioned above, the gold standard for diagnosis of LAM is made by histopathological findings. However, as in this case, decision to pursue and timing of surgical treatment is patient-specific. CT and MRI imaging remain the most accessible modalities for diagnosing lymphadenopathy in the absence of surgical exploration. Nonetheless, this case also highlights the appropriate controversy that exists regarding the accuracy of identifying lymph node metastasis from endometrial cancer solely by integrated imaging techniques, which more recently also include PET-CT imaging [15, 21]. In diseases which may mimic metastasis associated with gynecologic malignancy, we think PET-CT may offer substantial advantages in distinguishing key pathological feature of lymphadenopathy. However, slightly increased FDG uptake, as seen in this case, may cause difficulty in accurate characterization of the underlying abnormality. A review of more cases, further examining how to best evaluate pelvic and para-aortic lymph node metastasis or LAM in patients with comorbid endometrial cancer, is warranted to clarify the optimal diagnostic modality for early detection of sporadic LAM.

## Abbreviations

APS, anti-phospholipid antibody syndrome; CT, computed tomography; E2, estrogen; ER, estrogen receptor; FIGO, federation of gynecology and obstetrics; HMB, human melanin black; ITP, idiopathic thrombocytopenic purpura; LAM, lymphangioleiomyomatosis; mPSL, methylprednisolone; MRI, magnetic resonance imaging; mTOR, mammalian target of rapamycin; PEC, perivascular epithelioid cells; PET-CT, positron emission tomography-computed tomography; PIK3CA, phosphatidylinositol-4,5-bisphosphate 3-kinase, catalytic subunit alpha; PTEN, phosphatase and tensin homolog; SLE, systemic lupus erythematosus; SMA, smooth muscle actin; SUV, standardized uptake value; TSC, tuberous sclerosis complex
